# Non-invasive brain stimulation associated mirror therapy for upper-limb rehabilitation after stroke: Systematic review and meta-analysis of randomized clinical trials

**DOI:** 10.3389/fneur.2022.918956

**Published:** 2022-07-19

**Authors:** Qingqing Zhao, Hong Li, Yu Liu, Haonan Mei, Liying Guo, Xianying Liu, Xiaolin Tao, Jiang Ma

**Affiliations:** ^1^Department of Rehabilitation Medicine, Shijiazhuang People's Hospital, Shijiazhuang, China; ^2^College of Nursing and Rehabilitation, North China University of Science and Technology, Tangshan, China; ^3^Faculty of Graduate Studies, Hebei Medical University, Shijiazhuang, China

**Keywords:** stroke, mirror therapy (MT), non-invasive brain stimulation (NIBS), upper motor function, repetitive transcranial magnetic stimulation (rTMS), transcranial direct current stimulation (tDCS), meta-analysis

## Abstract

**Background:**

Non-invasive brain stimulation (NIBS) techniques and mirror therapy (MT) are promising rehabilitation measures for stroke. While the combination of MT and NIBS has been employed for post-stroke upper limb motor functional rehabilitation, its effectiveness has not been examined.

**Objective:**

This study aimed to evaluate the effectiveness of combined MT and NIBS in the recovery of upper limb motor function in stroke patients.

**Methods:**

The search was carried out in PubMed, EMBASE, Cochrane Library, Web of Science, Science Direct, CNKI, WANFANG and VIP until December 2021. Randomized clinical trials (RCTs) comparing MT or NIBS alone with the combination of NIBS and MT in improving upper extremity motor recovery after stroke were selected. A meta-analysis was performed to calculate the mean differences (MD) or the standard mean differences (SMD) and 95% confidence intervals (CI) with random-effect models. Subgroup analyses were also conducted according to the types of control group, the types of NIBS, stimulation timing and phase poststroke.

**Results:**

A total of 12 articles, including 17 studies with 628 patients, were reviewed in the meta-analysis. In comparison with MT or NIBS alone, the combined group significantly improved body structure and function (MD = 5.97; 95% CI: 5.01–6.93; *P* < 0.05), activity levels (SMD = 0.82; 95% CI 0.61–1.02; *P* < 0.05). For cortical excitability, the motor evoked potential cortical latency (SMD = −1.05; 95% CI:−1.57–−0.52; *P* < 0.05) and the central motor conduction time (SMD=-1.31 95% CI:−2.02-−0.61; *P* < 0.05) of the combined group were significantly shortened. A non-significant homogeneous summary effect size was found for MEP amplitude (SMD = 0.47; 95%CI = −0.29 to 1.23; *P* = 0.23). Subgroup analysis showed that there is an interaction between the stimulation sequence and the combined treatment effect.

**Conclusion:**

In this meta-analysis of randomized clinical trials, in comparison to the control groups, MT combined with NIBS promoted the recovery of upper extremity motor function after stroke, which was reflected in the analysis of body structure and function, activity levels, and cortical excitability.

**Systematic review registration:**

https://www.crd.york.ac.uk/prospero/, identifier CRD42022304455.

## Introduction

From 1990 to 2019, the global burden of stroke increased significantly. The absolute number of stroke incidence increased by 70.0%, deaths from stroke increased by 43.0%, and disability-adjusted life-years (DALYs) due to stroke increased by 32.0%. Stroke remained the second-leading cause of death and the third-leading cause of death and disability globally (1). Furthermore, approximately 80% of stroke patients have residual limb dysfunctions, of which upper limb dysfunction is one of the most common comorbidities (2). Upper limb impairment is generally considered to be persistent and disabling (3). More than half of patients with upper limb dysfunction will still suffer from this condition many months to years after stroke, which significantly reduce the ability of patients to live independently (4). Therefore, improving upper limb function is a core element of post-stroke rehabilitation.

Many possible interventions have been developed to help patients maximize the recovery of upper extremity function. In a pilot study, Altschuler et al. (5) found that mirror therapy (MT) may provide a beneficial adjunctive treatment for stroke patients. In this therapy, visual feedback is employed to stimulate the motor cortex of the cerebral hemisphere and activate the mirror neuron system to improve brain plasticity. Several recent reviews and meta-analyses have concluded that, compared with other physical practice, MT has a better therapeutic efficacy on upper limb motor function and activities of daily living in stroke patients, primarily with improvements in the Fugl-Meyer Assessment Upper Extremity (FMA-UE) and the Modified Barthel Index (MBI) (6–8). On the other hand, non-invasive brain stimulation (NIBS) has been widely used in clinical practice. Its therapeutic effect has also been affirmed. NIBS can modulate the cortical excitability and neuroplasticity of cerebral cortical neurons (9). The two most common techniques used for NIBS are repetitive transcranial magnetic stimulation (rTMS) and transcranial direct current stimulation (tDCS) (10).

Recently, many studies have combined MT with tDCS, and MT with rTMS, which represents a promising therapeutic approach for post-stroke upper limb functional rehabilitation (11, 12). Nevertheless, there are no systematic reviews and meta-analyses to confirm the synergistic effect of MT and NIBS in upper limb rehabilitation in stroke patients. Therefore, this systematic review and meta-analysis aims to evaluate the efficacy of combined MT and NIBS in the rehabilitation of upper limb motor function in stroke patients in comparison with the application of MT or NIBS alone.

## Materials and methods

The study was conducted with the PRISMA Protocol (13) for systematic reviews of randomized controlled trials. The protocol has been registered on PROSPERO (registration number CRD42022304455).

### Search strategy and selection criteria

We searched in the following databases for relevant literature: PubMed, EMBASE, Cochrane Library, Web of Science, Science Direct, CNKI, WANFANG and VIP. All articles up to December 2021 have been searched without any country, language, or article type restrictions. Reference lists of all selected articles were independently screened to identify additional studies missed in the initial search. The following search terms were used: “stroke,” “cerebrovascular accident,” “upper limb,” “hand function,” “mirror therapy,” “mirror visual feedback,” “rehabilitation,” “hemiparesis,” “non-invasive brain stimulation,” “TMS,” “rTMS,” and “tDCS.”

Two authors independently screened studies according to the inclusion and exclusion criteria. First, the titles and abstracts of the articles were read to exclude articles that clearly did not meet the inclusion criteria. Subsequently, by reading the full text, the final decision on whether to include the study was made. The differences between the two authors were resolved by consensus. A third examiner was invited to assist the article selection process if necessary.

### Eligibility criteria

Study participants include adult patients with upper extremity dysfunction caused by stroke. Randomized controlled trials (RCTs) in which active NIBS was added before, during, or after MT to improve the prognosis of upper limb were included. Two types of NIBS were evaluated: tDCS and rTMS. Studies using NIBS and MT alone, or in combination with each other's sham stimulation as controls were included. With regard to outcome measures, studies with the following items in reference to the International Classification of Functioning, Disability and Health (ICF) (2) were included: (a) body structure/function domain [e.g., Fugl Meyer Assessment Upper Extremity (FMA-UE)]; (b) activity levels [e.g., Modified Barthel Index (MBI), Action Research Arm Test (ARAT), Box and Blocks Test (BBT)]; (c) Neurophysiological Indicators [e.g., the amplitude of the motor evoked potential (MEP), cortical latency of MEP (MEP-CL), and central motor conduction time (CMCT)]. Articles with sample sizes of fewer than 10 participants and those with incomplete data were excluded.

### Data extraction and quality assessment

Two authors independently extracted data from each selected study. When disagreement occurs, a third author assisted the team to reach consensus. The following information was extracted: study characteristics (authors, year of publication, countries, sample sizes, age, gender, disease course, affected limbs and types of stroke) and intervention details (intervention measures, treatment time, stimulated sites, treatment parameters, and outcome measures). We also analyzed possible adverse events. In addition, if more than one outcome for activity levels exists, we select the outcome in the following order: MBI > ARAT > BBT.

Authors assessed the risk of bias using the Cochrane's Risk of Bias tool (14), including the terms of adequate sequence generation, allocation concealment, blinding, incomplete outcome data, selective reporting of the outcome, and other sources of bias. Rare scoring discrepancies were resolved after discussion with the third author. Results of bias assessment were presented in a figure and a graph indicating low, high, or unclear risk of bias for each of the 6 items in each trial.

### Statistical analysis

The outcomes in both the treatment and control groups after the intervention period were extracted. Quantitative synthesis was performed using RevMan 5.4. *P* values ≤ 0.05 were considered statistically significant. All outcomes in this study were continuous variables. For studies in which the same scale was used to evaluate the outcome, mean difference (MD) with 95% confidence interval (CI) were calculated. For outcomes assessed by different scales (e.g., activity levels), standardized mean difference (SMD) with 95% CI were analyzed. Since there are many differences in the measurement of motor evoked potentials with transcranial magnetic stimulators among the selected studies, such as coil type, stimulation intensity, target muscle location, we calculated SMD with 95% CI. We assessed heterogeneity among included studies using the *I*^2^ statistical method, in which significant heterogeneity is indicated for *I*^2^ > 50%. In cases of moderate or high heterogeneity, to confirm whether our findings were not driven by a single study, a leave-one-out sensitivity analysis was also performed by iteratively removing one study at a time. Funnel plot analysis was performed to evaluate the potential for publication bias for meta-analysis. Regardless of the magnitude of heterogeneity, we used a random-effect model for our analysis because we included studies of tDCS or rTMS.

If there were two combined NIBS and MT treatment groups (e.g., in different order) or two control groups (e.g., NIBS alone and MT alone) in an article, we separated this article into two independent studies. The targeted intervention of our study was the combination of MT and NIBS, which is the experimental arm in all analyses. We segmented the results according to the types of control group and established two distinct subgroups for comparison: MT and NIBS. In addition, subgroup analyses were also conducted according to the types of NIBS, stimulation timing and phase poststroke. Forest plots were used to represent the results of the meta-analysis.

## Results

### Search results

**Figure 1:**
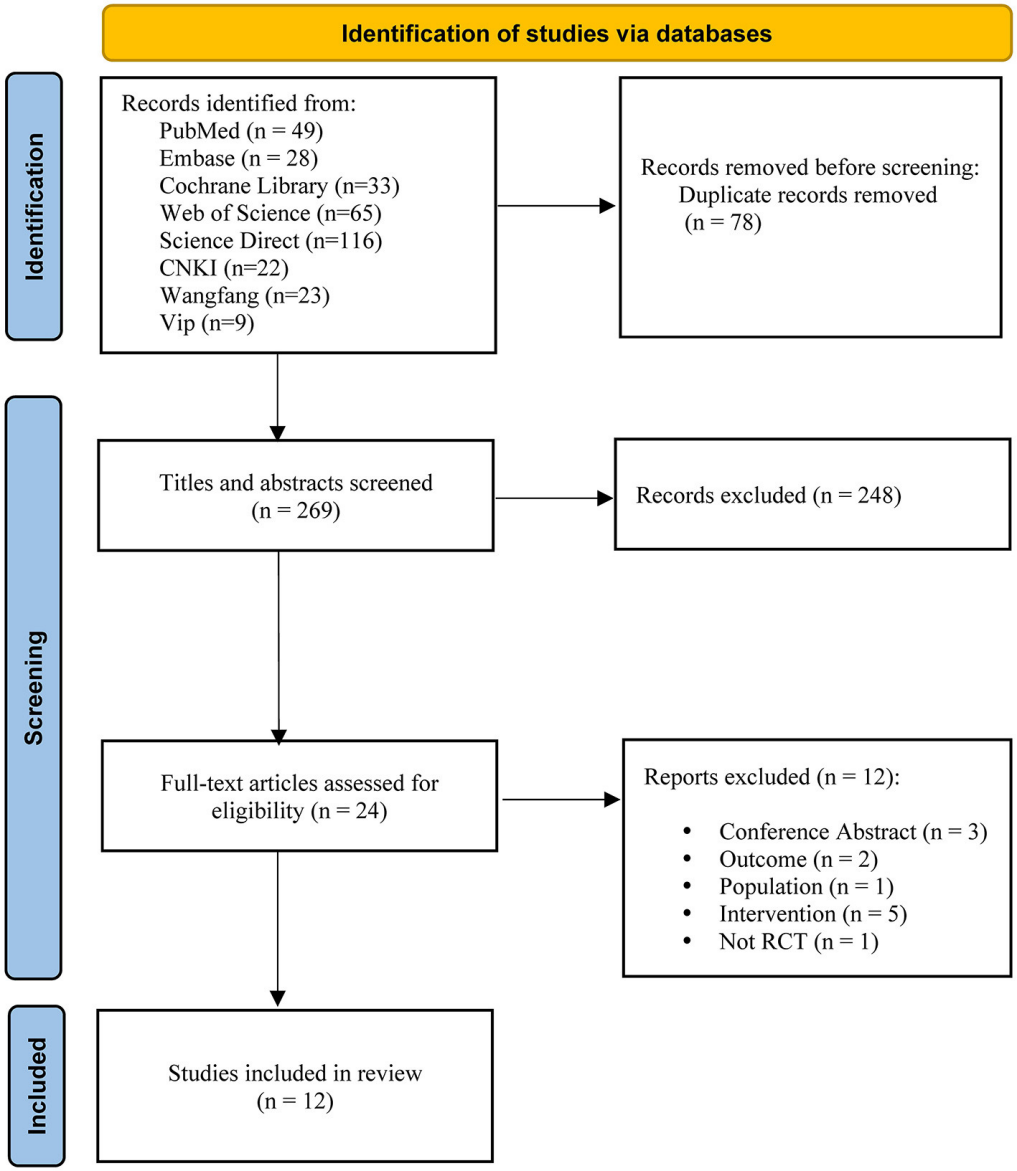
Flow diagram of the search process and study selection.

The search strategy proposed retrieved a total of 347 articles. After removing duplicates, the number was reduced to 269. A total of 248 articles were excluded by reading the title and abstract, and another 24 were selected for full-text reading. Finally, this systematic review and meta-analysis included 12 articles with a total of 628 patients. The Figure 1 depicts the flow diagram.

### Characteristics of the trials

The characteristics of participants in the trials selected for this systematic review and meta-analysis are shown in Table 1 (11, 12, 15–24). The characteristics of interventions are shown in Table 2. The age range of the study subjects is 51–74 years. All participants were in the subacute or chronic post-stroke phase.

**Table 1:** Key characteristics of the studies included in the systematic review.

**Reference**	**Country**	**Treatment**	**Number of patients (M/F)**	**Age (Mean** ±**SD)**	**Months/weeks/ days post-stroke (Mean** ±**SD)**	**Type of stroke (I/H)**	**Affected limb (L/R)**	**NIBS**	**Therapy protocol**	**Outcome measures**
Jil et al. (15)	Korea	I: MT + rTMS C: MT C2: Sham MT	I: 12 (7/5) C: 11 (7/4) C2: 12 (8/4)	I: 54.73 ± 7.88 C: 50.53 ± 8.02 C2: 52.45 ± 8.08	I: 9.20 ± 4.01, Mo C: 8.23 ± 3.09, Mo C2: 9.23 ± 4.29, Mo	I: 7/5 C: 6/5 C2: 6/5	I:4/8 C:5/6 C2:5/7	High-frequency (10 Hz) rTMS, ipsilesional	I: 15-min MT, 15 min of 10 Hz rTMS on the hotspot of the lesional hemisphere; C: 15-min MT; C2: 15-min sham therapy after covering the mirror with a white cloth. Intervention: 5 sessions/week for 6 weeks.	FMA-UE; BBT; MEP-CL; MEP amplitude
Cho and Cha (16)	Korea	I: Prior-tDCS + MT C: tDCS + sham MT	I: 14 (8/6) C: 13 (7/6)	I: 58.29 ± 10.67 C: 60.38 ± 10.19	I: 13.2 ± 5.1, Mo C: 15.5 ± 7.8, Mo	I: 9/5 C: 8/5	I: 8/6 C: 6/7	Anodal tDCS, ipsilesional, Anode: C3 and C4,Cathode: supraorbital area of the non-paretic side, 2 mA	I: 20-min anodal tDCS, 5-min rest, 20-min MT; C: 20-min anodal tDCS, 20-min sham therapy after covering the mirror with a white cloth. Intervention: 3 sessions/week for 6 weeks	FMA-UE; BBT; Jebsen-Taylor test; Grip strength
Liu et al. (18)	China	I: Prior-rTMS + TOMT C: TOMT C2: Conventional treatment	I: 30 (16/14) C: 30 (14/16) C2: 30 (17/13)	I: 58.30 ± 14.65 C: 56.00 ± 14.23 C2: 58.63 ± 13.45	I: 128.73 ± 4.71, D C: 133.87 ± 26.01, D C2: 133.50 ± 24.23, D	I: 30/0 C: 30/0 C2: 30/0	I: 10/20 C: 11/19 C2: 12/18	low-frequency (1Hz) rTMS, contralesional	I: 15 min of 1 Hz rTMS on the hotspot of the contralesional hemisphere, followed by 30-min task-oriented MT; C: 30-min task-oriented MT; C2: Conventional treatment. Intervention: 6 sessions/week for 4 weeks	FMA-UE; MBI; MEP-CL; CMCT
Yang et al. (11)	China	I: Prior- MT + rTMS I2: Prior- rTMS + MT C: MT	I: 15 (15/0) I2: 14 (14/0) C: 15 (15/0)	I: 60.7 ± 12.3 I2: 55.3 ± 5.6 C: 57.2 ± 9.0	I: 60.7 ± 12.3, D I2: 105.2 ± 16.4, D C: 120.0 ± 33.3, D	I: 11/4 I2: 9/5 C: 10/5	I: 11/4 I2: 9/5 C: 10/5	High-frequency (10Hz) rTMS, ipsilesional	I: 50-min MT, followed by 10 min of 10 Hz rTMS on M1 of the lesional hemisphere; I2: 10 min of 10 Hz rTMS on M1 of the lesional hemisphere, followed by 50-minute MT; C: 60-min MT. Intervention: 5 sessions/week for 4 weeks	FMA-UE; MEP-CL; CMCT; MI; FTHUE-HK
Kim and Yim (17)	Korea	I: rTMS + TOMT C: rTMS	I: 8 (4/4) C: 12 (4/8)	I: 51 ± 2.98 C: 74.11 ± 2.88	I: 1.63 ± 0.74, Mo C: 1.75 ± 0.62, Mo	I: 7/5 C: 5/3	I: 5/7 C: 2/6	High-frequency (20Hz) rTMS, ipsilesional	I: 15 min of 20 Hz rTMS on the lesional hemisphere, 20-min task-oriented mirror therapy; C: 15 min of 20Hz rTMS on the lesional hemisphere. Intervention: 5 sessions/week for 2 weeks	BBT; Hand grip and pinch grip strength; RMT; MEP-CL; MEP amplitude
Li et al. (20)	China	I: Prior-rTMS + MT C: rTMS	I: 30 (17/13) C: 30 (16/14)	I: 55.4 ± 10.3 C: 56.8 ± 9.7	I: 24.7 ± 10.2, D C: 23.6 ± 11.8, D	I: 30/0 C: 30/0	I: 11/19 C: 12/18	low-frequency (1Hz) rTMS, contralesional	I: 20 min of 1Hz rTMS on the hotspot of the contralesional hemisphere, followed by 30-min MT; C: 20 min of 1 Hz rTMS on the hotspot of the contralesional hemisphere, followed by 30-min conventional occupational therapy. Intervention: 6 sessions/week for 4 weeks	FMA-UE; MI; MEP-CL; CMCT
Jin et al. (19)	China	I: Prior-tDCS + MT I2: Concurrent-tDCS + MT C: Sham-tDCS + MT	I: 10 (2/8) I2: 10 (3/7) C: 10 (1/9)	I: 59.00 ± 9.80 I2: 58.70 ± 7.92 C: 57.50 ± 7.08	I: 19.44 ± 8.25, Mo I2: 20.46 ± 11.13, Mo C: 22.16 ± 8.15, Mo	I: 8/2 I2: 4/6 C: 8/2	I: 6/4 I2: 2/8 C: 3/7	Bilateral tDCS; Cathodal: the contralesional hemisphere (C3), Anodal: the ipsilateral M1, 2 mA	I: 30-min bilateral tDCS, followed by 30-min MT; I2: Bilateral tDCS was applied for 30 min at the same time as the 30-min MT; C: Sham stimulation randomly prior to or concurrent with MT. Intervention: 5 sessions/week for 2 weeks	FMA-UE; ARAT; BBT
Chen et al. (21)	China	I: Prior- tDCS + MT C: MT C2: tDCS	I: 26 (13/13) C: 26 (10/16) C2: 26 (12/14)	I: 59.32 ± 8.59 C: 62.46 ± 7.92 C2: 61.31 ± 9.13	I: 58.92 ± 17.30, D C: 64.93 ± 16.02, D C2: 62.81 ± 18.14, D	I: 15/11 C: 12/14 C2: 13/13	I: 10/16 C: 9/17 C2: 8/18	Anodal tDCS, Anode: hotspot, Cathode: contralateral superior orbital rim, 2 mA	I: 20-min anode tDCS, followed by 30-min MT; C: 20-min anode tDCS; C2: 30-min MT. Intervention: 5 sessions/week for 3 weeks	FMA-UE; MBI; MEP-CL; CMCT
Wang (23)	China	I: Concurrent-tDCS + MT C: tDCS C2: MT C3: Conventional treatment	I: 30 (15/15) C: 30 (16/14) C2: 30 (17/13) C3: 30 (16/14)	I: 55.15 ± 7.30 C: 55.92 ± 7.66 C2: 53.30 ± 6.17 C3: 54.22 ± 8.26	10 days to 3 months	I: 16/14 C: 18/12 C2: 16/14 C3: 17/13	I: 13/17 C: 16/14 C2: 14/16 C3: 15/15	Anode tDCS, Anode: Ipsilateral cerebral hemisphere MI, Cathode: contralateral shoulder, 2 mA	I: Anode tDCS was applied for 20 min at the same time as the 20-min MT; C: 20-min anode tDCS; C2: 20-min MT; C3: Conventional treatment. Intervention: 5 sessions/week for 4 weeks	FMA-UE; MBI; ARAT; NIHSS
Liao et al. (22)	China	I: Prior- tDCS + MT C: Concurrent-tDCS + MT C2: Sham tDCS + MT	I: 8 (5/3) C: 12 (8/4) C2: 8 (8/0)	I: 60.18 ± 4.84 C: 52.04 ± 8.68 C2: 56.45 ± 9.88	I: 19.63 ± 12.28, Mo C: 21.92 ± 11.83, Mo C2: 38.13 ± 36.98, Mo	I: 6/2 C: 7/5 C2:7/1	I: 6/2 C: 7/5 C2: 8/0	Anode tDCS, Anodic: iM1, Cathodic: contralateral supraorbital region, 1–2 mA	I: 20-min anodal tDCS on iM1, followed by 20-min MT with sham tDCS and 20-min MT alone. C: 20 -min sham tDCS, followed by 20-min MT concurrently with anodal tDCS on iM1 and 20-min MT alone. C2: 20-min sham tDCS, followed by 20-min MT concurrently with sham tDCS and 20-min MT alone. Intervention: 5 sessions/week for 4 weeks	FMA-UE; NEADL: Kinematic Variables
Li (24)	China	I: rTMS + MT C: MT	I: 40 (22/18) C: 42 (23/19)	I: 59.10 ± 12.31 C: 57.60 ± 9.27	I: 31.64 ± 7.69, D C: 30.52 ± 7.19, D	NR	I: 22/18 C: 20/22	In the recovery phase, High-frequency (10Hz) rTMS, ipsilesional; In the acute phase, low-frequency (1 Hz) rTMS, contralesional	I: 15 min of 1 or 10 Hz rTMS on M1 of the contralesional or ipsilesional hemisphere, 20-min AM and 20-min PM MT; C: 20-min AM and 20-min PM MT. Intervention: 6 sessions/week for 3 weeks	FMA-UE; MBI; MEP-CL; CMCT; MAS; NIHSS
Yu and Chen (12)	China	I: tDCS + TOMT C: tDCS C2: Conventional treatment	I: 45 (22/23) C: 45 (24/21) C2: 45 (20/25)	I: 57.8 ± 11.2 C: 58.6 ± 12.3 C2: 60.4 ± 10.7	I: 62.9 ± 13.2, D C: 61.7 ± 12.6, D C2: 63.6 ± 11.6, D	I: 26/19 C: 28/17 C2: 31/14	I: 15/30 C: 18/27 C2: 16/29	Anodal tDCS, Anode: hotspot, Cathode: contralateral superior orbital rim, 2 mA	I: 20-min anodal tDCS on iM1, 40-min MT; C: 20-min anodal tDCS; C2: Conventional treatment. Intervention: 5 sessions/week for 6 weeks	FMA-UE; MBI; MEP-CL; CMCT

*AM, ante meridiem; ARAT, Action Research Arm Test; BBT, Box and Blocks Test; C, control; CL, cortical latency; CMCT, central motor conduction time; D, days; F, female; FMA-UE, Fugl Meyer Assessment Upper Extremity; FTHUE-HK, Functional Test For The Hemiplegic Upper Extremity – Hong Kong Version; H, hemorrhagic; I, ischemic; I, intervention; iM1, ipsilateral M1; L, left; M, male; MAS, Modified Ashworth Scale; MBI, modified Barthel Index; MEP, motor evoked potential; MI, motricity Index; Mo, months; MT, mirror therapy; NIBS, noninvasive brain stimulation; NIHSS, National Institute of Health stroke scale; NR, not reported; PM, post meridiem; R, right; RMT, resting motor threshold; rTMS, repetitive transcranial magnetic stimulation; SD, Standard Deviation; tDCS, transcranial direct current stimulation; TOMT, task-oriented mirror therapy*.

**Table 2:** Interventions reported in the meta-analysis.

**Reference**	**Participants, phase poststroke**	**NIBS**				**Type of control group**
		**NIBS paradigm**	**Timing of stimulation**	**Intensity or frequency of current**	**Number of sessions (daily)**	
Jil et al. (15)	Chronic	HF-rTMS on affected hemisphere	NR	10 Hz	30	MT
Cho and Cha (16)	Chronic	atDCS on affected hemisphere	Before MT	2 mA	18	tDCS
Liu et al. (18)	Chronic	LF-rTMS on unaffected hemisphere	Before MT	1 Hz	24	MT
Yang et al. (11)-A	Chronic	HF-rTMS on affected hemisphere	After MT	10 Hz	20	MT
Yang et al. (11)-B	Chronic	HF-rTMS on affected hemisphere	Before MT	10 Hz	20	MT
Kim and Yim (17)	Subacute	HF-rTMS on affected hemisphere	NR	20 Hz	10	rTMS
Li et al. (20)	Subacute	LF-rTMS on unaffected hemisphere	Before MT	1 Hz	24	rTMS
Jin et al. (19)-A	Chronic	Bilateral tDCS	Before MT	2 mA	10	MT
Jin et al. (19)-B	Chronic	Bilateral tDCS	During MT	2 mA	10	MT
Chen et al. (21)-A	Subacute	atDCS on affected hemisphere	Before MT	2 mA	15	tDCS
Chen et al. (21)-B	Subacute	atDCS on affected hemisphere	Before MT	2 mA	15	MT
Wang (23)-A	Subacute	atDCS on affected hemisphere	During MT	2 mA	20	tDCS
Wang (23)-B	Subacute	atDCS on affected hemisphere	During MT	2 mA	20	MT
Liao et al. (22)-A	Chronic	atDCS on affected hemisphere	Before MT	1–2 mA	20	MT
Liao et al. (22)-B	Chronic	atDCS on affected hemisphere	During MT	1–2 mA	20	MT
Li (24)	Subacute	LF-rTMS on unaffected hemisphere; HF-rTMS on affected hemisphere	NR	1 Hz; 10 Hz	18	MT
Yu and Chen (12)	Subacute	atDCS on affected hemisphere	NR	2 mA	30	tDCS

*atDCS, anodal tDCS; Chronic: ≥3months; HF, high frequency; LF, low frequency; MT, mirror therapy; NIBS, non-invasive brain stimulation; NR, not reported; rTMS, repetitive transcranial magnetic stimulation; subacute: 2 weeks- 3 months; tDCS, transcranial direct current stimulation*.

Among the 13 studies, there were three studies (11, 19, 22) that had two combined intervention groups. Each of the above studies is divided into two different studies (Yang J et al., A: Prior-MT + rTMS; Yang J et al., B: Prior-rTMS + MT; Jin MX et al., A and Liao WW et al., A: Prior-tDCS + MT; Jin MX et al., B and Liao WW et al., B: Concurrent-tDCS + MT). Two studies (21, 23) with two control groups were divided in two (Chen H et al., A: NIBS alone; Chen H et al., B: MT alone; Wang Q, A: NIBS alone; Wang Q, B: MT alone). Therefore, 12 publications with 17 studies were enrolled for the meta-analysis. With regard to NIBS, 7 studies used rTMS while 10 used tDCS. For control groups, 11 studies had MT alone while 6 studies had NIBS alone. For stimulation timing, 8 studies used NIBS before MT, 4 used NIBS during MT, and only one used NIBS after MT. The remaining studies did not specify the stimulation timing for both. For phase poststroke, 8 studies were completed in the subacute phase, 9 studies were completed in the chronic phase.

### Quality assessment

**Figure 2:**
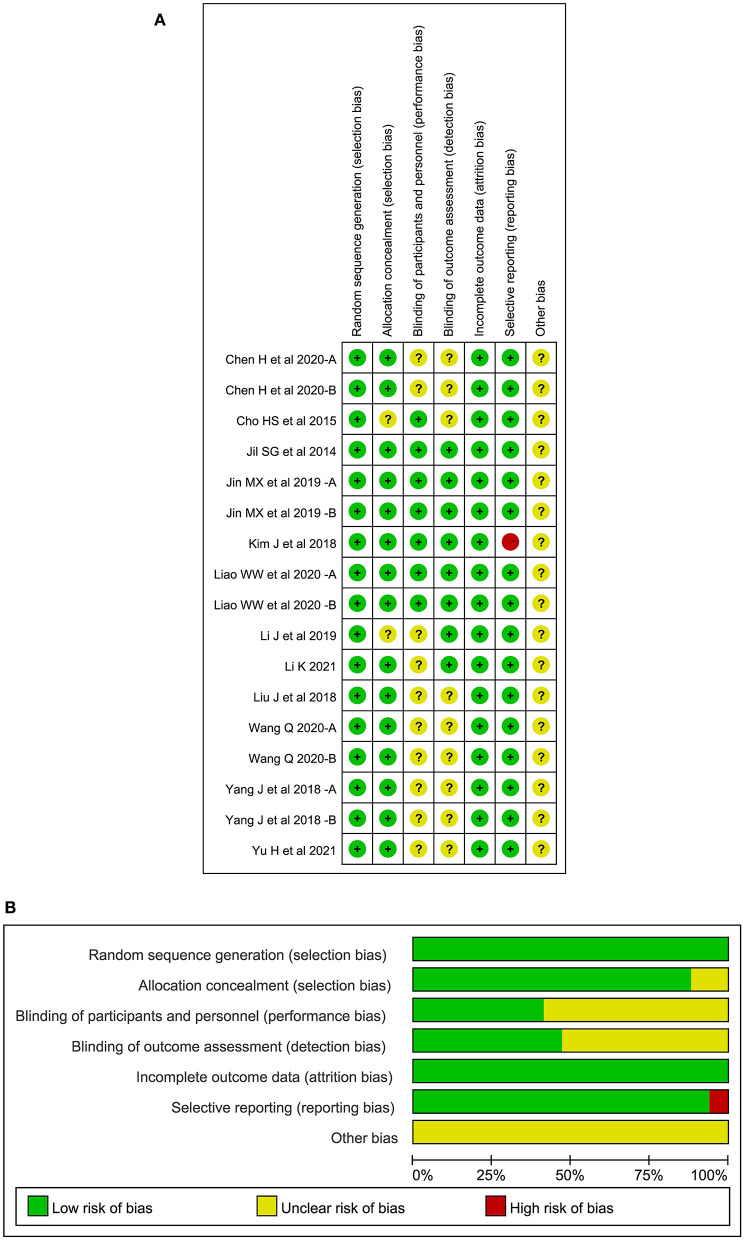
**(A)** Risk of bias summary, **(B)** Risk of bias graph.

All literatures mentioned the random grouping in the text. Fifteen studies of 10 articles reported appropriate random sequence generation methods, including random number table, computer random number system, and lottery. The remaining studies only mentioned the randomization and did not specify the specific grouping method. Fifteen studies of 10 articles reported the method of random allocation concealment while the rest did not mention this topic. Seven studies of 5 articles presented blinding of participants or personnel, 8 studies of 6 articles presented blinding of outcome assessment, and the rest did not specify blinding methods. One study had unbalanced missing data while the remaining studies reported complete outcomes. There was no selective reporting in all studies and other risk of bias could not be determined. The risk of bias of the included RCTs is shown in Figure 2. A sensitivity analysis showed that pooled effect size was not overaffected by the specific study, indicating the result was relatively robust.

### Publication bias

Funnel plots were generated for each observation index (Supplementary Figure 1). As can be seen from the funnel plots, the included studies were generally symmetric and concentrated, suggesting no substantial evidence of publication bias.

### Main analysis

#### Body structure/function domain

**Figure 3:**
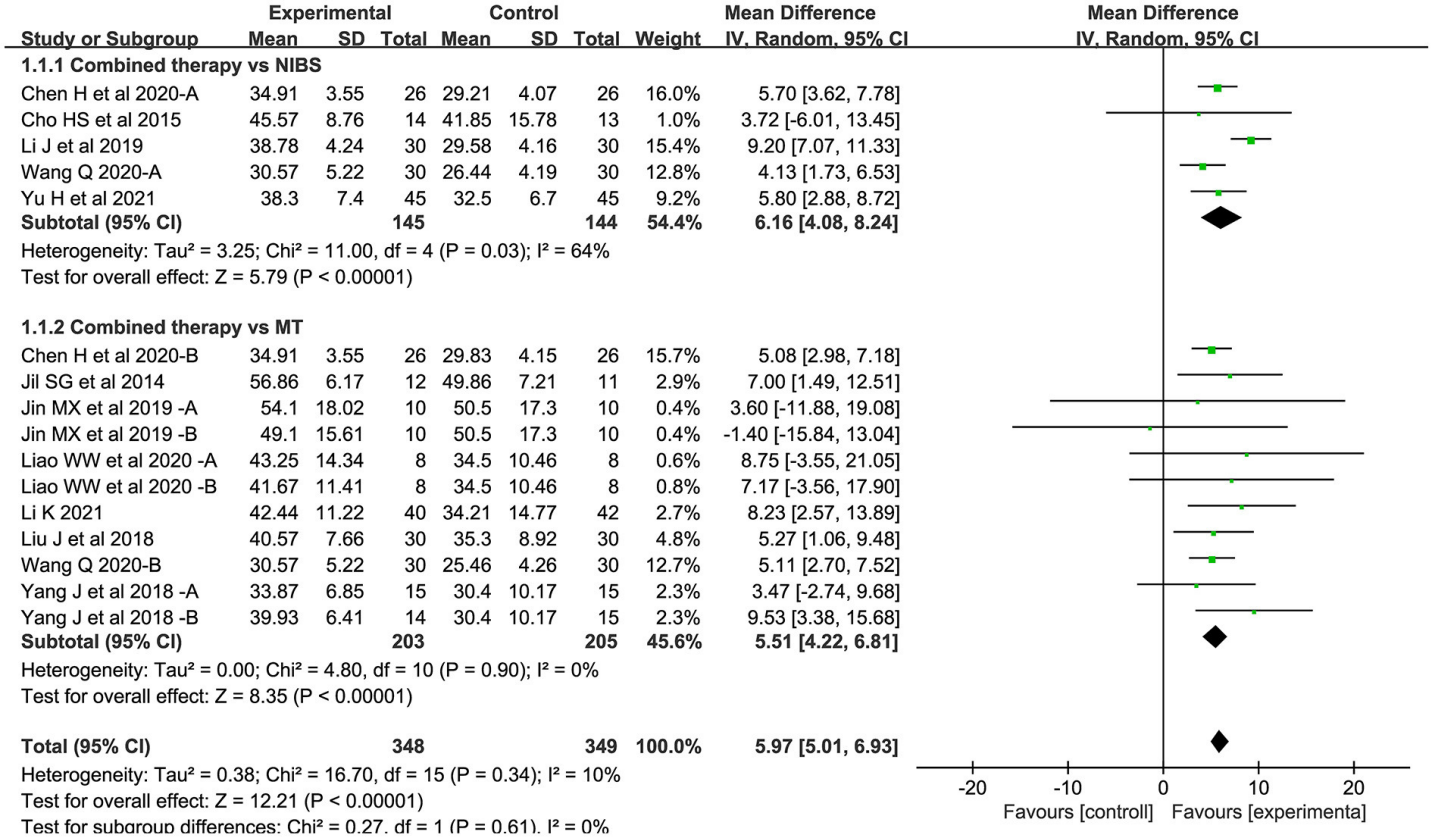
Forest plot of trails comparing NIBS combined with MT vs. NIBS or MT alone for body structure/functional domains.

Sixteen studies assessed the FMA-UE as an outcome measure of body structure/function domain. Overall, the combined intervention induced a significant improvement on body structure/function in comparison to the control group (MD = 5.97; 95% CI: 5.01–6.93; *P* < 0.05) (Figure 3). The forest plot shows that statistical heterogeneity was not observed (*I*^2^ = 10%; *P* = 0.34).

#### Activity levels

**Figure 4:**
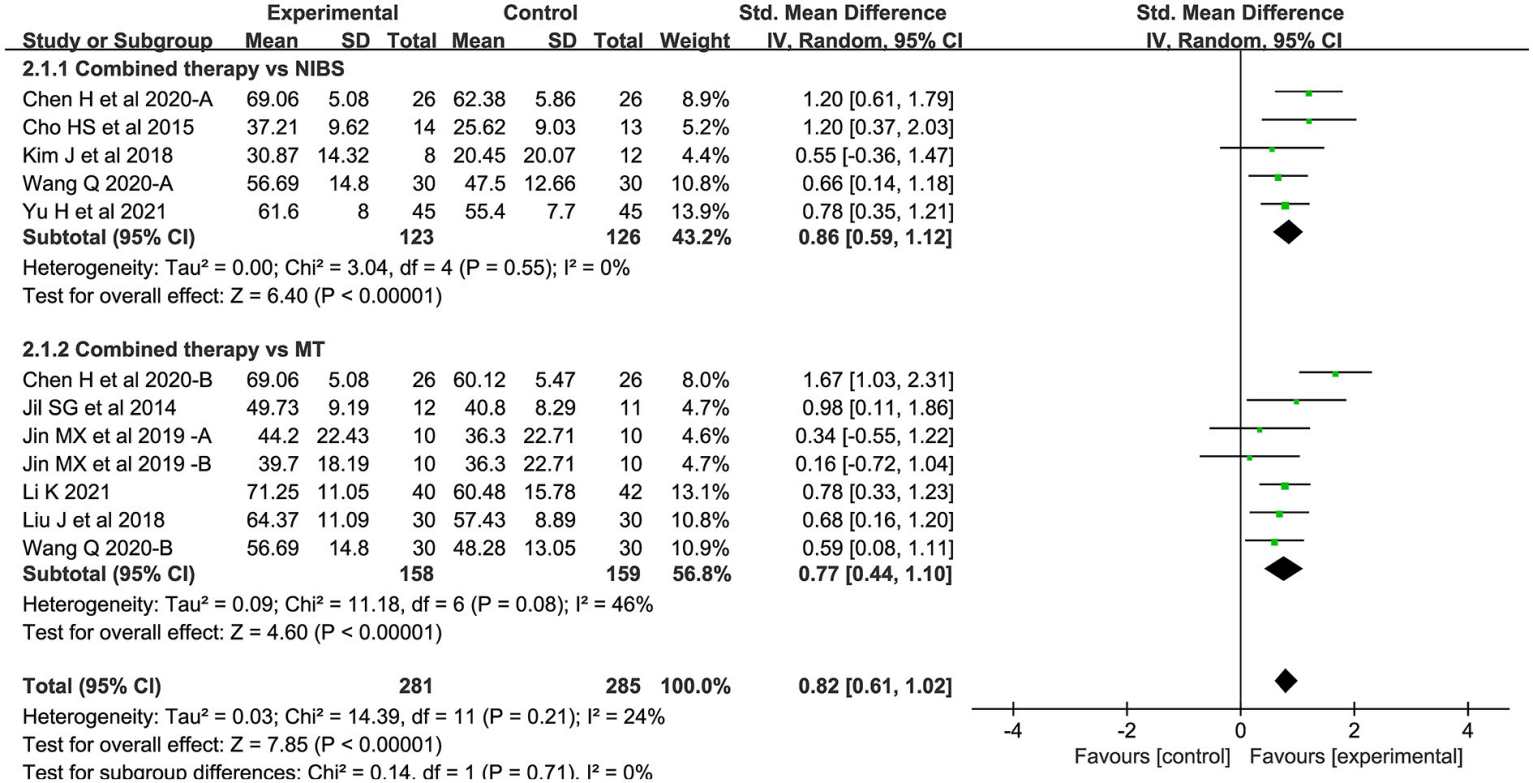
Forest plot of trails comparing NIBS combined with MT vs. NIBS or MT alone for activity levels.

Twelve studies assessed activity levels. Seven studies used MBI, 2 used ARAT, and 3 used BBT. Overall, the combined intervention induced a significant improvement on activity levels in comparison to the control group (SMD = 0.82; 95% CI: 0.61–1.02; *P* < 0.05) (Figure 4). The forest plot shows that statistical heterogeneity was not observed (*I*^2^ = 24%; *P* = 0.21).

#### Neurophysiological indicators

##### MEP-CL

**Figure 5:**
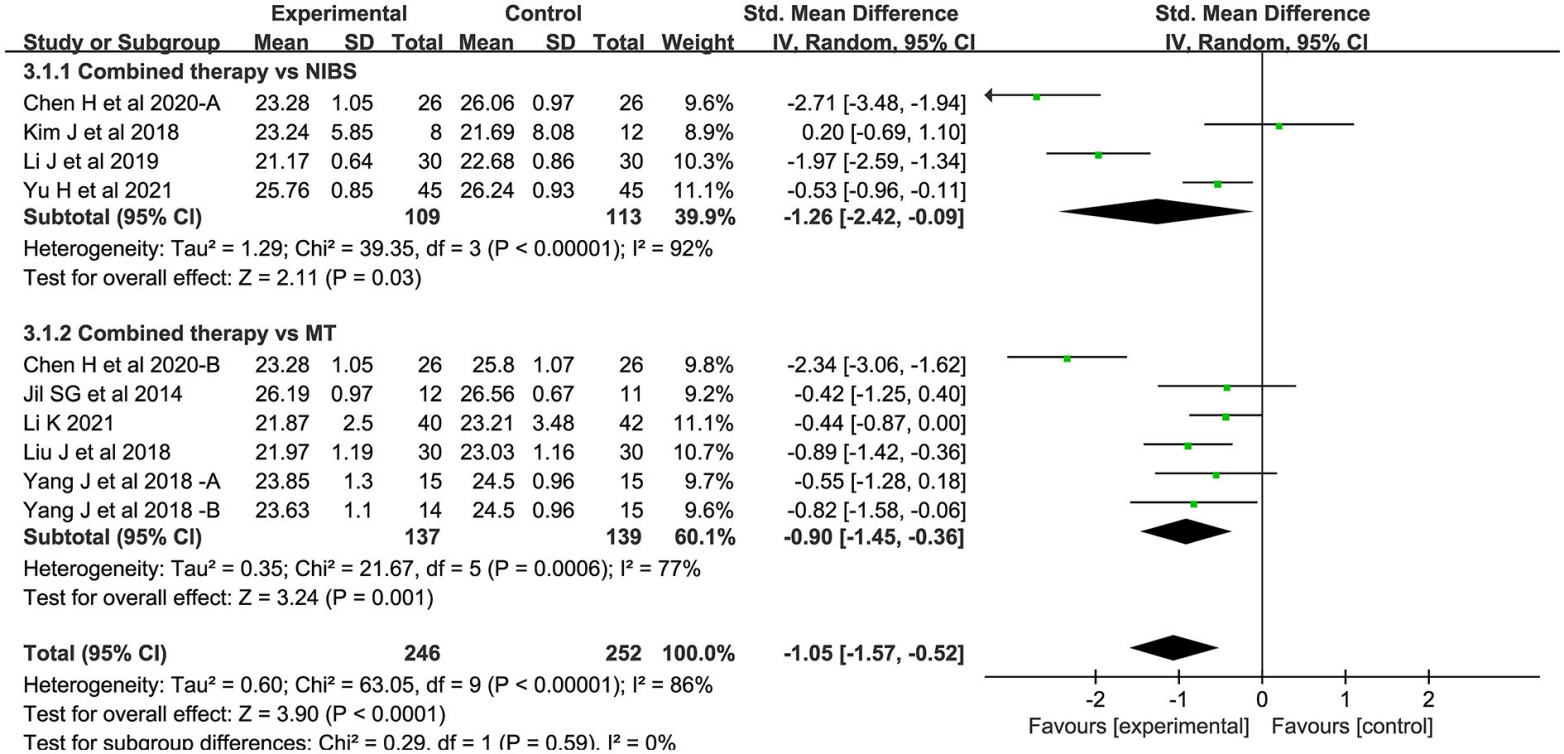
Forest plot of trails comparing NIBS combined with MT vs. NIBS or MT alone for motor evoked potential cortical latency [MEP-CL].

Ten studies measured motor evoked potential cortical latency. Compared to the control group, the latency of the combined intervention group was significantly shorter (SMD = −1.05; 95% CI:−1.57-−0.52, *P* < 0.05) (Figure 5). There was significant heterogeneity between studies (*I*^2^ = 86%; *P* < 0.05).

##### CMCT

**Figure 6:**
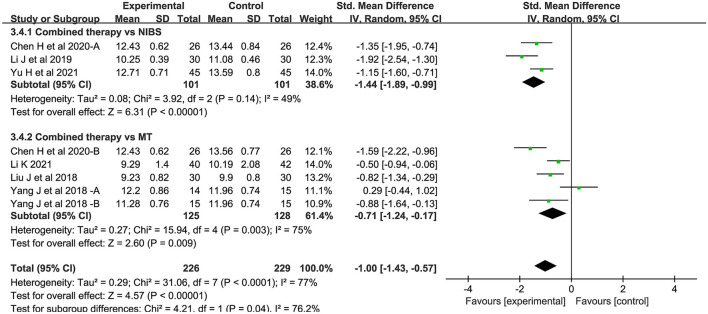
Forest plot of trails comparing NIBS combined with MT vs. NIBS or MT alone for central motor conduction time [CMCT].

Eight studies measured central motor conduction time. Compared with the control group, the CMCT of the combined intervention group was significantly shortened (SMD = −1.00; 95% CI:−1.43-−0.57; *P* < 0.05) (Figure 6). There was significant heterogeneity between studies (*I*^2^ = 77%; *P* < 0.05).

##### MEP amplitude

**Figure 7:**
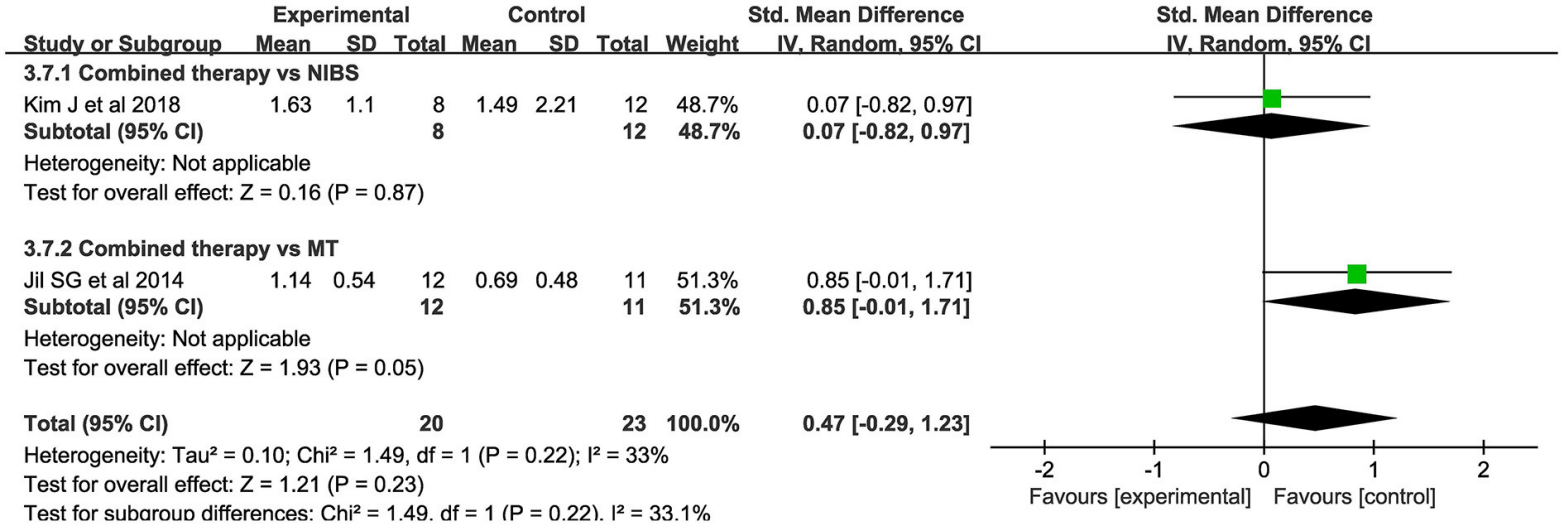
Forest plot of trails comparing NIBS combined with MT vs. NIBS or MT alone for motor evoked potential [MEP] amplitude.

Only 2 studies evaluated MEP amplitude. NIBS combined with MT did not modify the amplitude of MEPs when compared to the control group (SMD = 0.47; 95% CI:−0.29–1.23; *P* > 0.05) (Figure 7). There was no significant heterogeneity (*I*^2^ = 33%; *P* = 0.22).

##### Adverse events

Li (24) reported that one patient in the intervention group (rTMS combined with MT) developed epilepsy. Cho and Cha (16) reported that 2 headache patients dropped out of the study during tDCS application. Kim and Yim (17) reported that 4 patients in the intervention group (HF-rTMS combined with TOMT) dropped out due to scalp pain or personal reasons. We do not know whether these adverse events are related to their experiment designs. No adverse reactions were reported in other articles.

### Subgroup analysis

**Figure 8:**
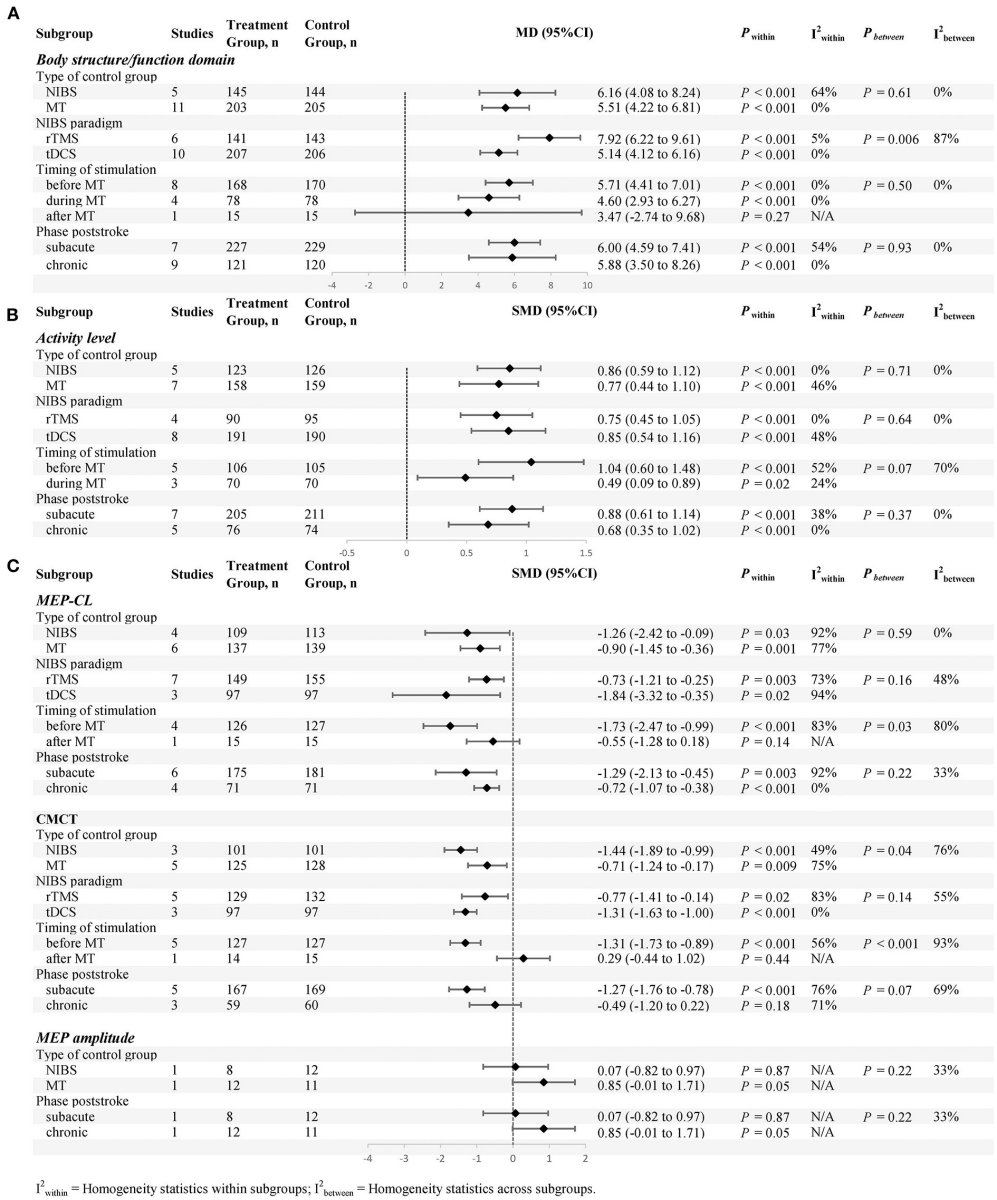
Subgroup analyses (defined according to the types of control group, NIBS paradigm, stimulation time and phase poststroke) are shown, **(A)** is body structure/functional domain, **(B)** is activity levels, **(C)** is neurophysiological Indicators.

Subgroup analyses (defined according to the types of control group, NIBS paradigm, stimulation time and phase poststroke) are shown in Figure 8.

#### Body structure/function domain

For the types of control group, we found significant results for both subgroups: (1) NIBS alone (MD = 6.16; 95% CI: 4.08–8.24; *P* < 0.05; *I*^2^ = 64%) and (2) MT alone (MD = 5.51; 95% CI: 4.22–6.81; *P* < 0.05; *I*^2^ = 0%).

For NIBS paradigm, the results were significant for both rTMS (MD = 7.92; 95% CI: 6.22–9.61; *P* < 0.05; *I*^2^ = 5%) and tDCS (MD = 5.14; 95% CI: 4.12–6.16; *P* < 0.05; *I*^2^ = 0%).

For stimulation timing, we found significant results in both the first two subgroups: (1) before MT (MD = 5.71; 95% CI: 4.41–7.01; *P* < 0.05; *I*^2^ = 0%) and (2) during MT (MD = 4.60; 95% CI: 2.93–6.27; *P* < 0.05; *I*^2^ = 0%). No significant results were found in the last subgroup: (3) after MT (MD = 3.47; 95% CI:−2.74–9.68; *P* = 0.27).

For phase poststroke, the results were significant for the subacute (MD = 6.00; 95% CI: 4.59–7.41; *P* < 0.05; *I*^2^ = 54%) and chronic (MD = 5.88; 95% CI: 3.50–8.26; *P* < 0.05; *I*^2^ = 0%). The above results regarding body structure/function domain are shown in (Figure 8A).

#### Activity levels

For the types of control group, we found significant results for both subgroups: (1) NIBS alone (SMD = 0.86; 95% CI: 0.59–1.12; *P* < 0.05; *I*^2^ = 0%) and (2) MT alone (SMD = 0.77; 95% CI: 0.44–1.10, *P* < 0.05, *I*^2^ = 46%).

For NIBS paradigm, the results were significant for both rTMS (SMD = 0.75; 95% CI: 0.45–1.05; *P* <0.05; *I*^2^ = 0%) and tDCS (SMD = 0.85; 95% CI: 0.54–1.16; *P* < 0.05; *I*^2^ = 48%).

For stimulation timing, we found significant results in both subgroups: (1) before MT (SMD = 1.04; 95% CI: 0.60–1.48; *P* < 0.05; *I*^2^ = 52%) and (2) during MT (SMD = .49; 95% CI: 0.09–0.89; *P* < 0.05; *I*^2^ = 24%).

For phase poststroke, the results were significant for the subacute (SMD = 0.88; 95% CI: 0.61–1.14; *P* < 0.05; *I*^2^ = 38%) and chronic (SMD = 0.68; 95% CI: 0.35–1.02; *P* < 0.05; *I*^2^ = 0%). The above results regarding activity levels are shown in (Figure 8B).

#### Neurophysiological indicators

For the types of control group, we found that MEP-CL in both subgroups were significantly shortened: (1) NIBS alone (SMD = −1.26; 95% CI:−2.42-−0.09; *P* < 0.05; *I*^2^ = 92%) and (2) MT alone (SMD = −0.90; 95% CI:−1.45-−0.36, *P* < 0.05; *I*^2^ = 77%). Likewise, CMCT was significantly improved in both groups: (1) NIBS alone (SMD = −1.44; 95% CI:−1.89-−0.99; *P* < 0.05; *I*^2^ = 49%) and (2) MT alone (SMD = −0.71; 95% CI:−1.24-−0.17; *P* < 0.05; *I*^2^ = 75%). However, we found no significant difference in MEP amplitude, whether compared with rTMS alone (SMD = 0.07; 95% CI:−0.82–0.97; *P* = 0.87), or MT alone (SMD = 0.85; 95% CI:−0.01–1.71; *P* = 0.05).

For NIBS paradigm, we found that MEP-CL in both subgroups were significantly shortened: (1) rTMS (SMD = −0.73; 95% CI:−1.21-−0.25; *P* < 0.05; *I*^2^ = 73%) and (2) tDCS (SMD = −1.84; 95% CI:−3.32-−0.35; *P* < 0.05; *I*^2^ = 94%). Likewise, CMCT was significantly improved in both groups: (1) rTMS (SMD = −0.77; 95% CI:−1.41-−0.14; *P* < 0.05; *I*^2^ = 83%) and (2) tDCS (SMD = −1.31; 95% CI:−1.63-−1.00; *P* < 0.05; *I*^2^ = 0%).

For stimulation timing, a statistically significant effect on both subgroups was present before MT: (1) MEP-CL (SMD = −1.73; 95% CI:−2.47-−0.99; *P* < 0.05; *I*^2^ = 83%) and (2) CMCT (SMD = −1.31; 95% CI:−1.73-−0.89; *P* < 0.05; *I*^2^ = 56%). Conversely, no statistically significant effect on both subgroups was present after MT: (1) MEP-CL (SMD = −0.55; 95% CI:−1.28–0.18; *P* = 0.14) and (2) CMCT (SMD = 0.29; 95% CI:−0.44–1.02; *P* = 0.44).

For phase poststroke, MEP-CL in both subgroups were significantly shortened: (1) subacute (SMD = −1.29; 95% CI:−2.13-−0.45; *P* < 0.05; *I*^2^ = 92%) and (2) chronic (SMD = −0.72; 95% CI:−1.07-−0.38; *P* < 0.05; *I*^2^ = 0%). There was a significant decrease in the CMCT during the subacute phases (SMD = −1.27; 95% CI:−1.76-−0.78; *P* < 0.05; *I*^2^ = 76%). In chronic phases, however, this phenomenon was not observed (SMD = −0.49; 95% CI:−1.20–0.22; *P* = 0.18; *I*^2^ = 71%). For MEP amplitude, no statistically significant effect on both subgroups was present: (1) subacute (SMD = 0.07; 95% CI:−0.82–0.97; *P* = 0.87), and (2) chronic (SMD = .85; 95% CI:−0.01–1.71; *P* = 0.05). The above results regarding neurophysiological indicators are shown in (Figure 8C).

## Discussion

Twelve articles were included in this meta-analysis. Since some articles were divided into two studies, a total of 17 studies of 628 patients were included in the analysis. We analyzed the effect of combined treatment of MT and NIBS on the recovery of upper extremity motor function in stroke patients. Comparing combination therapy to NIBS or MT alone to evaluate a real synergistic effect has important clinical implications (25). In view of the results, we concluded that the combined treatment of NIBS and MT is more effective than single treatments for the recovery of upper extremity motor function after stroke. Similar results were observed by Luo et al. (26). Despite of the high heterogeneity, their results indicated that combining MT with another rehabilitation therapy was more effective than single rehabilitation therapies for the upper extremity of stroke patients. Contrary to our findings, Saavedra-Garcia et al. (27) concluded that the combination of MT with electrical stimulation resulted in similar improvements in upper extremity motor function relative to MT alone. However, the difference between these studies and the present study is that the combined effect of MT and NIBS was not detected. Similarly, a recent meta-analysis showed that NIBS combined with other therapies was effective in improving upper extremity motor function and activities of daily living in acute/subacute stroke patients, but not in chronic stroke patients (28). In contrast, a meta-analysis by Reis et al. (29) indicated that there were not enough data about the benefits of NIBS as an additional intervention to robotic-assisted therapy on upper extremity motor function or activity in stroke patients. Therefore, to the best of our knowledge, this study is the first meta-analysis summarizing the findings on MT combined with NIBS in upper limb motor function recovery after stroke.

The results of this study were divided into three categories: evaluation of body structure/function, activity levels and cortical excitability. We used FMA-UE, the most frequently employed scale to evaluate the therapeutic effect of upper limb motor function after stroke, to evaluate body structure/function (30). Compared to the control group, the FMA-UE score of the combined treatment group was significantly improved, indicating an enhancement of the upper limb motor function of patients in the combined treatment group. In terms of activity levels (such as ADL/BBT/ARAT), seven studies evaluated MBI, two studies evaluated ARAT and three studies evaluated BBT. Similar to FMA-UE changes, these scores also showed better improvement in the combined treatment group. In these two results, no significant heterogeneity was observed, which increased the reliability of the results. Using neuroelectrophysiological indicators to predict functional recovery is very important in stroke. Transcranial magnetic stimulation is a non-invasive means to evaluate the structure and function of the central nervous system, such as MEP amplitude and latency, and central motor conduction time (CMCT), provides insight into the corticospinal excitability status after stroke (31, 32). In this study, ten studies measured the MEP-CL of the affected side, and eight studies measured CMCT. Compared with the single intervention, MEP-CL and CMCT were significantly shortened after combined intervention. The excitability of cerebral cortex was improved. Only two studies evaluated the amplitude of the motor evoked potential. NIBS combined with MT was not effective to improve the MEP amplitude. This is inconsistent with other results that reflect the excitability of the cerebral cortex. A small sample size may be one of the reasons for this result, and further in-depth research is needed to expand the sample size. In addition, the heterogeneity of these three indicators was high (33–94%). The heterogeneity of MEP measurement may result from two reasons (33): internal heterogeneity, which is the internal fluctuation of excitability of cortical and spinal cord neurons between repeated stimulation processes in the same patient, and inter-individual heterogeneity, which is the heterogeneity between different subjects due to coil position, age, and drug intake. Although transcranial magnetic stimulation is considered a potential evaluation method, its reliability remains to be further investigated. Future studies should avoid controllable differences as much as possible and reduce heterogeneity in meta-analysis.

By providing normal visual stimulation of bilateral movement, MT has been proven to be effective in enhancing upper limb motor function in stroke patients by many clinical studies (8, 34, 35). Among the non-invasive brain stimulation techniques, the most widely studied are rTMS and tDCS. rTMS as a neuro-stimulator and tDCS as a neuro-modulator can promote motor recovery by activating or inhibiting activity in cortical areas, particularly when combined with appropriate behavioral interventions (36, 37). An emerging area of clinical research is the development of NIBS and MT combination, with the expectation that this approach will maximize the efficacy of single therapies. As shown in our results, the combined treatment of MT with NIBS significantly improved body structure/function and activity levels, and significantly shortened MEP-CL and CMCT relative to the single treatments, despite of the higher heterogeneity in MEPs. In a subgroup analysis, we also observed that the combination treatment was superior to the known levels of NIBS alone or MT alone. We infer that the combination of NIBS and MT might enhance synaptic plasticity and induce neurogenesis in order to improve motor performance.

NIBS can directly regulate the excitability of stroke damaged cortex and restore the balance between bilateral cerebral hemispheres. For example, high-frequency rTMS and anodal tDCS increased motor cortex excitability in the affected brain while low-frequency rTMS and cathodal tDCS inhibited the contralateral cortex and indirectly activated the affected cortex. Based on the “ -peripheral-central” closed-loop model proposed by Jia Jie's research group, MT organically combines central and peripheral intervention to form a “closed-loop” information feedback, thereby activating the mirror neuron system and the main motor cortex and promoting central remodeling and peripheral control, and ultimately improving the motor function of the upper extremity in post-stroke patients (38). The remodeling of brain function by MT needs to go through additional nerve conduction pathways, which can be activated by NIBS, resulting in a shortening of MT-mediated mobilization of mirror neurons and an improvement in the efficiency of MT-mediated mirror neuron activation and neural network remodeling (11). In light of the results of this meta-analysis, we propose that NIBS, as an exogenous neuromodulation, and MT, an endogenous neuronal activation, combine to produce a more stable and potent enhancement of cortical excitability. This hypothesis warrants further clinical and experimental validation. In a previous study, Di Lazzaro et al. (39) combined tDCS or sham stimulation with Constraint-Induced Movement Therapy and found that the two groups performed similarly on behavioral measures. They also measured MEP in each group by TMS and found that the intervention group significantly increased cortical excitability relative to the control group. However, this alteration was insufficient to induce behavioral changes. Unlike conventional rehabilitation therapies, MT allows patients to have less motor output. In NIBS, patients even have no motor output. However, the results here showed that even without high-intensity and long-term peripheral intervention, the combined intervention of MT and NIBS not only improved the excitability of the patient's cerebral cortex but also performed significantly better than the single intervention groups in various functional indexes (MBI, ARAT, etc.).

With regard to the effects of combination therapy, there are key factors to consider, such as the type of NIBS, the timing of stimulation of combination therapy, and the poststroke phase. The most ideal treatment for stroke patients is to directly stimulate the central nervous system (40). rTMS has a mechanism similar to tDCS, which can promote the recovery of upper limb motor function by regulating the abnormal excitability of bilateral brains after stroke. Most studies and clinical applications have proved that rTMS has a certain effect (41, 42). We found that MT, whether combined with tDCS or rTMS, significantly improved FMA-UE score, activity levels (e.g., ADL/BBT/ARAT), and cortical excitability (e.g., MEP-CL, CMCT). This finding is consistent with a previous observation on the effect of tDCS combined with physical training on cortical excitability (37). Moreover, we also found statistically significant differences between rTMS and tDCS subgroups on the forest plot of body structure/function. Therefore, the types of NIBS showed a significant interaction with the effects of combination therapy (*P* = 0.006). Contrary to our results, the meta-analysis by Ahmed et al. (28) showed that in comparison to tDCS sham stimulation, tDCS combined with other treatments had a positive effect on FMA-UE scores. However, rTMS combined with other treatments showed no significant improvement in comparison to single therapies. TMS can induce neurons to generate action potentials, and by stimulating the complete motor cortex of the efferent pathway, the corresponding muscle contractions produce corresponding actions, but tDCS cannot produce this effect. Ka et al. (43) demonstrated that there was no statistically significant difference between rTMS and tDCS in motor performance. Larger studies are required to elucidate the superiority of both approaches. In fact, in clinical applications, it can be selected according to its specific conditions and the differences between the two NIBS.

Another key factor to consider in the evaluation of the effectiveness of the combined therapy is the stimulation time of NIBS intervention relative to MT. Jin et al. (19) and Liao et al. (22) obtained opposite conclusions when they observed the intervention effects of tDCS before MT or at the same time on upper limb motor function in chronic stroke patients. In our subgroup analysis, we found that in comparison to single treatments, NIBS before and during MT showed significant improvement in FMA-UE scores and activity levels, and there was no significant difference between subgroups. Kang et al. (44) also obtained similar conclusions, which could be explained by the widely recognized priming stimulation mechanism. This theory suggests that “the brain that has been primed by prior activation is generally more responsive to the accompanying or subsequent training” and assumes that enhanced neural activity before or during training can promote the activation of long-term enhancement (LTP) or long-term inhibition (LTD) (45). Therefore, this theory suggests that NIBS before or during MT can improve the upper limb motor function of stroke patients. In the studies included in this meta-analysis, rTMS and MT were not performed simultaneously because the patient's head needed to be kept in a fixed position during this period, and was therefore used before or after mirror therapy. Further analysis revealed that in FMA-UE and activity levels, the effect size of tDCS before MT was slightly higher than that of the simultaneous application of both. A previous study suggested that the simultaneous application of NIBS and MT may produce motor/cognitive interference during MT training in order to inhibit motor relearning and limit the generalization of motor skills (22). We speculate that the combination of NIBS and MT do not simply additive effects and its outcomes may depend on the stimulation sequence. In the future, it is necessary to discuss this issue in conjunction with the excitatory or inhibitory mechanisms of NIBS to further clarify the optimal stimulation sequence and its underlying mechanisms.

On the other hand, the analysis showed that NIBS before MT significantly improved FMA-UE scores and also shortened the cortical latency and central motor conduction time of motor evoked potential, which was not observed NIBS after MT. NIBS is a stimulation-based priming while bilateral mirror symmetric motion is a movement-based priming (45). These techniques can be used as the primary interventions or as a priming method for follow-up exercise training (45). In this study, we observed that stimulus-based priming seemed to be more effective. As mentioned before, we analyzed that MT is a repetitive bilateral symmetrical movement, which requires the patient's movement initiative and needs to go through additional nerve conduction pathways. Therefore, NIBS, as an exogenous neuromodulation, provides a precondition for MT. Similarly, rTMS priming of subsequent physical therapy was found to be optimal by Avenanti et al. (46). Additional clinical and experimental studies are necessary to support this theory in the future.

We define priming as a session of neural conditioning that modifies the aftereffects of a subsequent conditioning session, with the effect often described in terms of metaplasticity (47). Over a period, neural network regulation caused by two consecutive events may change brain plasticity in unexpected ways-either facilitation or inhibition (48). Such changeable plasticity (i.e., the plasticity of synaptic plasticity) is known as metaplasticity. According to a recent review, facilitatory training after facilitation priming may lower synaptic activity in the subsequent regulatory process, diminishing the aftereffects of the latter, which diminished learning while preserving the synapse (metaplasticity) (48). On the other hand, training after inhibitory priming strengthens the effects of motor learning (49). How different priming regimens influence the induction effect of a test regimen on corticospinal excitability in healthy individuals was investigated in a meta-analysis by Hassanzahraee et al. (50). The results suggest that, especially if synaptic activity is high or low enough to change the threshold, a history of high synaptic activity favors inhibition or a history of low synaptic activity favors facilitation. Only two of the studies in this meta-analysis used inhibitory priming stimulation, and accepted thinking advises against using subgroup analyses to make firm conclusions when covariate distribution is unequal. This is a limitation of the research, and the following research should focus on the effects of different priming stimulations on the treatment of stroke patients. Future treatment plans will be based on the combination of brain stimulation techniques and physical therapy. In order to choose the most effective combination therapy, more clinical trials are needed to determine the time-dependent effects of the therapy.

In this meta-analysis, patients in the subacute or chronic stages were included. As a result, subgroup analyses were carried out according to poststroke phase. At both stages, there were significant improvements in activity levels, MEP-CL, and body structure or function, with no significant between-group differences. The CMCT of patients in the chronic phase was shown to have not significantly improved (*P* > 0.05). The two subgroups' amplitudes did not significantly improve. According to recent studies, rTMS, tDCS, and MT have some therapeutic effects in the subacute and chronic stages of stroke, although their results from simple application are poor (42, 51). Additionally, the early stages of recovery seem to be quite promising for this combined therapy. NIBS and MT are now rarely used in the acute phase of stroke. Rehabilitation as soon as possible when patients can tolerate it is the concept that experts in the field of rehabilitation have always advocated for (2). Since the early post-stroke period is an effective window for neuroplasticity enhancement, rehabilitation may be particularly effective during this period. Both MT and NIBS do not depend on the patient's residual ability. These approaches can also be utilized early, providing a full-course, simple and integrated intervention program for clinical rehabilitation.

There are some limitations in this systematic review. First, the number of studies was small, including five articles in English and seven articles in Chinese. Second, there is a high heterogeneity in neuroelectrophysiological indexes, which limits the generalizability of the findings. Third, the NIBS protocol has inconsistencies, such as stimulation frequency or target spot, current intensity, treatment time, etc. In the future, researchers need to analyze the effectiveness of combined treatment according to these parameters. In addition, it is necessary to carry out medium-term and long-term follow-up to further evaluate the effectiveness of combination therapy.

## Conclusion

The current review explored the therapeutic effects of the combination of MT and NIBS. The results suggest that MT combined with NIBS could promote the recovery of upper limb motor function in stroke in three aspects: body structure and function, activity levels, and cortical excitability. In addition, this review also discussed several issues: (1) the comparison of combination therapy with NIBS or MT alone to evaluate potential synergy; (2) the comparison of the effects of MT in combination with rTMS or tDCS; (3) the optimal stimulation timing of NIBS (before, during, or after MT); and (4) the comparison of the effects of combined therapy in different courses of disease (subacute or chronic). Although there are some limitations, we believe that the findings and the recommendations provided in this review may help to select the most appropriate combination protocol to maximize the recovery of upper limb motor function in stroke patients.

## Data availability statement

The original contributions presented in the study are included in the article/Supplementary Material, further inquiries can be directed to the corresponding author.

## Author contributions

QZ and HL: literature search, data extraction, and manuscript drafting. YL and HM: statistical analysis. QZ and XT: quality evaluation. XL and LG: typographical logic of the article. JM and HL: design of the study and commentary on important intellectual content. All authors contributed to the article and approved the submitted version.

## Funding

This study was funded by the S&T Program of Hebei (2037727D).

## Conflict of interest

The authors declare that the research was conducted in the absence of any commercial or financial relationships that could be construed as a potential conflict of interest.

## Publisher's note

All claims expressed in this article are solely those of the authors and do not necessarily represent those of their affiliated organizations, or those of the publisher, the editors and the reviewers. Any product that may be evaluated in this article, or claim that may be made by its manufacturer, is not guaranteed or endorsed by the publisher.
