# Supplementary material for: Non-invasive brain stimulation associated mirror therapy for upper-limb rehabilitation after stroke: Systematic review and meta-analysis of randomized clinical trials
Source: Front Neurol. 2022 Jul 19;13:918956. doi: 10.3389/fneur.2022.918956 (PMC9345505; doi:10.3389/fneur.2022.918956)
Supplement: Supplementary file 1 [file Data_Sheet_1.docx]

Supplementary Material

## Supplementary Figures

**
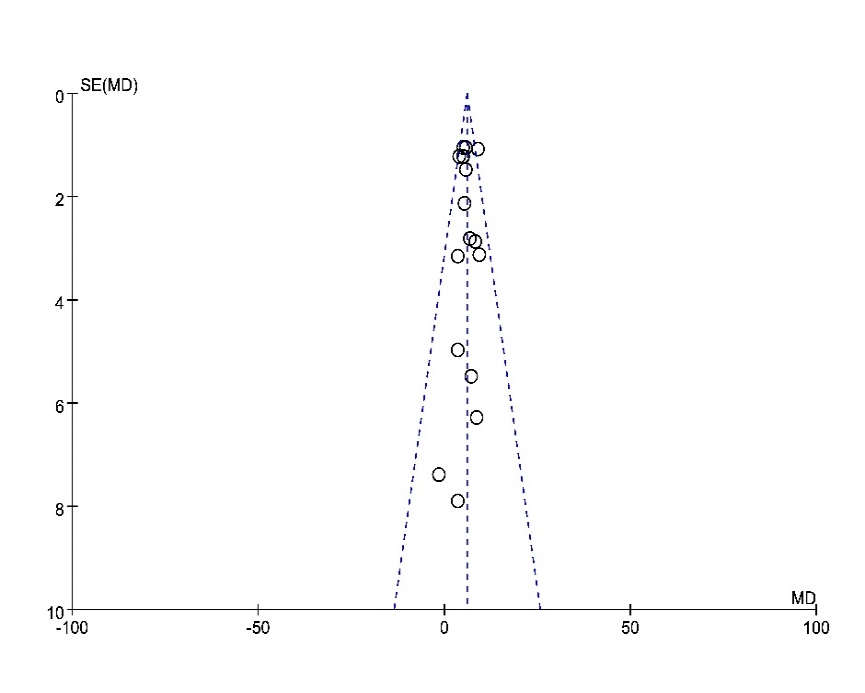
**

**A**


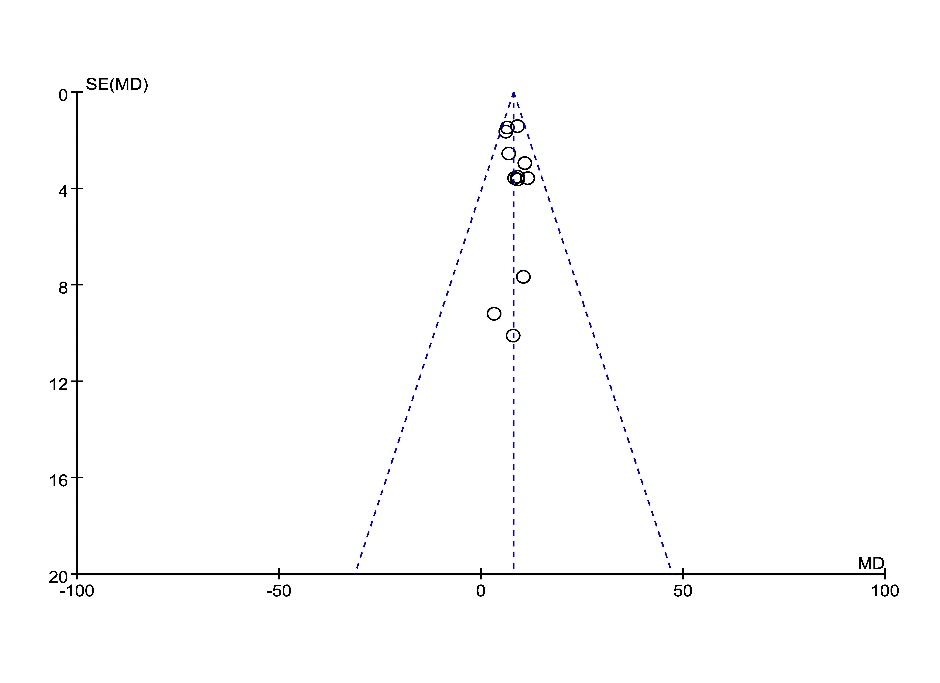


**B**

**
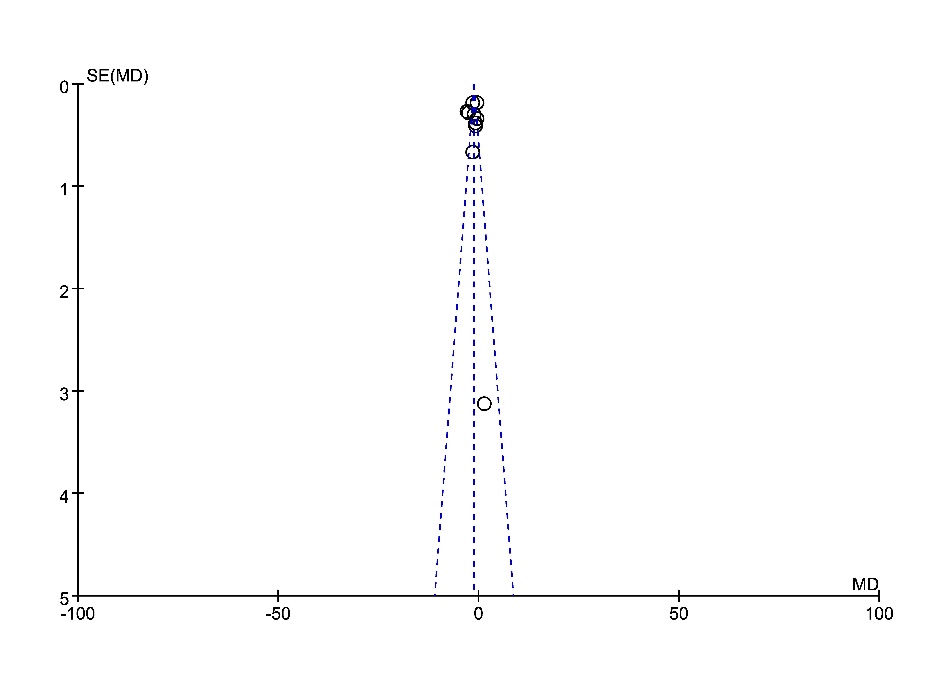
C**


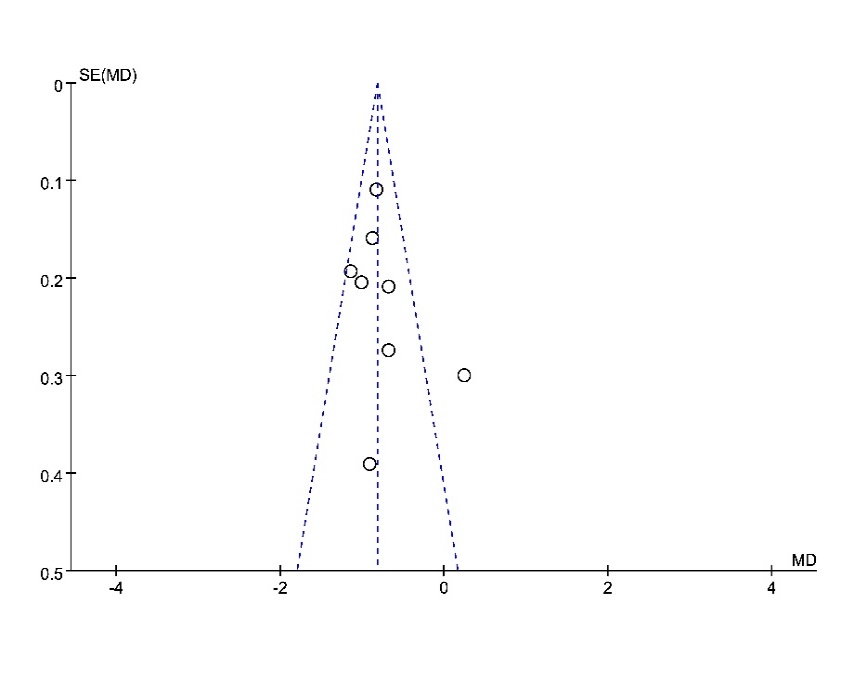


**D**


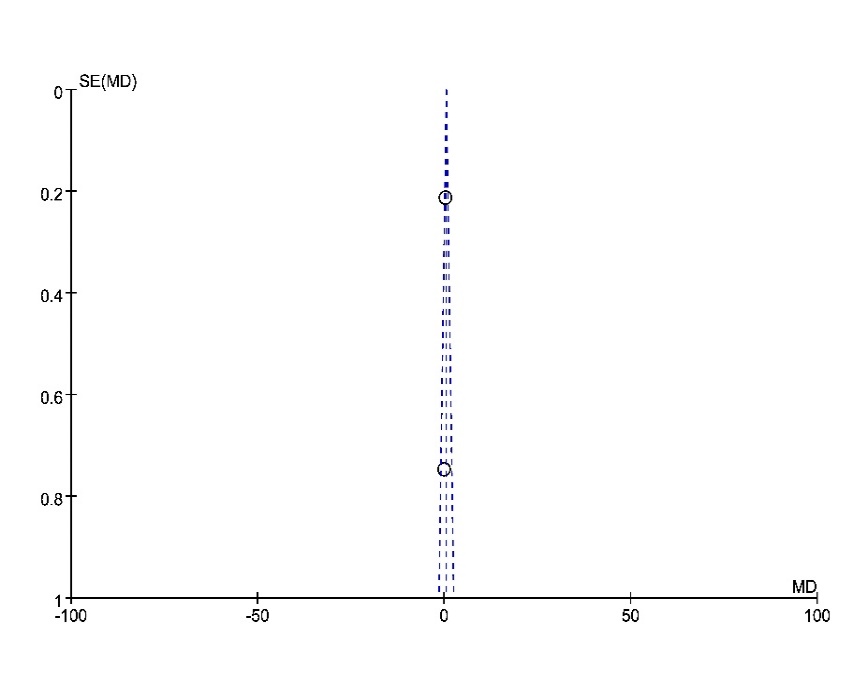


**E**

**Supplementary Figure 2.** The funnel plots assesses publication bias in the included studies, (**A**) is body structure/function domain, (**B**) is activity levels, (**C**) is MEP-CL, (**D**) is CMCT, (**E**) is MEP Amplitude.
